# COVID-19 related deaths in an urban academic medical center in Brooklyn – a descriptive case series

**DOI:** 10.1186/s41231-020-00065-y

**Published:** 2020-08-13

**Authors:** James Andrew McCracken, Mohamed Nakeshbandi, Jeffrey Arace, Wayne J. Riley, Roopali Sharma

**Affiliations:** University Hospital of Brooklyn, SUNY Downstate Health Sciences University, 450 Clarkson Avenue, Box 36, Brooklyn, New York 11203 USA

**Keywords:** Coronavirus, COVID-19, Mortality, SARS-CoV-2

## Abstract

**Background:**

Available studies are lacking in analysis of baseline demographics and hospital presentation of patients at risk of expiring due to coronavirus disease 2019 (COVID-19), particularly Black American patients. We conducted a retrospective chart review to determine similarities in demographics and hospital presentation among patients who expired due to COVID-19 at an academic medical center in Brooklyn, New York.

**Study design and methods:**

This is a retrospective observational study of 200 patients who expired due to complications of COVID-19. Patients were included in this study if they had laboratory-confirmed SARS-CoV-2 infection and expired during their admission. Data were collected on patients who expired between March 17 and April 16, 2020.

**Results:**

A vast majority of patients were Black Americans (89%) with no history of international travel who had more than one comorbidity (81%), with the most common comorbidities being hypertension (84·5%), diabetes mellitus (57·5%), and obesity (41·5%). Fifty-five percent of our patient population had three or more comorbidities. Among patients with available data, C-reactive protein, lactate dehydrogenase, and ferritin values were elevated above normal limits at admission. Dyspnea was the most common presenting symptom (92·5%). Most (90·5%) presented within the first week of symptoms, with a median time of symptoms prior to expiration being 8·42 days (IQR 5·57–12·72).

**Interpretation:**

Socioeconomic status and healthcare inequalities have greatly affected the Black population of Brooklyn, New York, and these disparities become even more apparent in COVID-19 infection. Patients presenting with numerous comorbidities and elevated inflammatory markers represent a population at high risk of in-hospital mortality.

## Background

In December 2019, China reported a cluster of cases of novel coronavirus disease 2019 (COVID-19) caused by severe acute respiratory syndrome coronavirus 2 (SARS-CoV-2) [1]. Since then COVID-19 has become a global pandemic. As of April 26, 2020, 3,012,387 cases and 207,885 deaths have been reported worldwide [2]. The first reported case in the United States (US) occurred on January 19, 2020 in Washington state [3], and more than 993,000 COVID-19 cases have been reported since in the US, with approximately 55,729 deaths [2]. New York state has become the epicenter of this pandemic in the US, with more than 291,000 cases and approximately 17,300 deaths reported as of April 26 [2], making up over one-quarter of total US cases. Over 156,000 cases had been reported in New York City (NYC) alone, with approximately 12,000 confirmed deaths across five boroughs [4].

Although COVID-19 has spread across the country, its impact on the diverse populations of the US is far from equitable. A disproportionate burden of cases of COVID-19 has been reported in Black Americans, affecting these populations at an exceptionally high rate. COVID-19 related illness is much more severe and twice as deadly in these minorities compared to their white counterparts, with Black Americans accounting for 34% of deaths (population representation: 29%) [4]. In Chicago, more than 50% of COVID-19 cases and nearly 70% of deaths involve Black Americans, despite Black Americans making up only 30% of the population. Moreover, these deaths are concentrated in five neighborhoods on Chicago’s South Side [5]. In Louisiana and Michigan, the majority of COVID-19 deaths have occurred in Black Americans, contrasting with Black Americans’ low representation in these states’ populations [6, 7]. Of reported race data for fatalities in NYC, Black Americans make up 127·1 cases per 100,000 deaths, compared to 63·5 cases per 100,000 of deaths in white patients [8].

In Brooklyn, there have been 41,327 confirmed cases of COVID-19 as of April 26, with more than 3494 deaths – one of the highest COVID-19 related fatality rates among the boroughs of NYC [8]. One commonality in the data reported from Brooklyn, Chicago, Louisiana, and Michigan is that these areas are home to lower-income and immigrant populations. These hard-hit areas highlight how the pandemic reflects and exacerbates health disparities and inequalities between neighborhoods. Because of such disparities, Black Americans are contracting SARS-CoV-2 at higher rates and are more likely to die [9]. Higher rates of mortality in Black Americans may also be associated with risk factors such as hypertension, cardiovascular disease, diabetes, and obesity. Many reports have documented that COVID-19 illness is more severe in patients with these pre-existing conditions [10–13]. In this paper we describe the demographics, baseline comorbidities, presenting laboratory values, and in-hospital course and outcomes of 200 patients with COVID-19 who died in an academic healthcare facility in Brooklyn, New York.

## Methods

### Study design and population

This retrospective descriptive case series included inpatients ≥18 years of age who expired due to COVID-19 at University Hospital of Brooklyn (UHB) at State University of New York (SUNY) Downstate Health Sciences University, the only academic medical center in Brooklyn. UHB was designated as a COVID-19-only facility by New York Governor Andrew Cuomo in late March 2020. Patients were included in this study if they had documented positive SARS-CoV-2 RNA serology tests. Expirations in this study occurred between March 17 and April 16, 2020.

### Data collection

Data were collected from the Allscripts® Sunrise™ platform. Data reviewed included, but were not limited to: baseline demographics (age, sex, race, weight, body mass index (BMI)); laboratory values (complete blood count and basic metabolic panel); inflammatory markers (C-reactive protein (CRP), ferritin, lactate dehydrogenase (LDH), D-dimer, fibrinogen, erythrocyte sedimentation rate (ESR)); past medical history; and medication profiles. Baseline laboratory values at admission were reviewed. Final values at expiration were reviewed if bloodwork was performed within 24 h of expiration. Patients were excluded from evaluation of a laboratory value if the value was not available. Past medical history (including comorbidities and medication history) and symptoms associated with COVID-19 were recorded based on documentation in the admission profile by admitting physicians. To account for patients who were admitted to the hospital for chief complaints other than COVID-19 who subsequently tested positive, the duration of symptoms was adjusted based on when symptoms associated with COVID-19 began.

### Definitions

Fever was defined as temperature greater than 37·3 **°**C. Lymphocytopenia was defined as absolute lymphocyte count < 900 cells/mcL. Acute kidney injury (AKI) was defined based on available serum creatinine (SCr) data in one of three ways: using Kidney Disease Improving Global Outcomes staging criteria, where Stage 1 AKI is an increase in SCr 1·5–1·9 times baseline, Stage 2 AKI an increase of 2.0–2.9 times baseline, and Stage 3 AKI an increase of 3 or more times baseline; or the initiation of hemodialysis (utilizing admission SCr and expiration SCr to calculate trend); or, for patients without available SCr trend indicating AKI, baseline SCr more than 1·5 times the upper limit of normal (ULN) (i.e., > 1·95 mg/dL) or blood urea nitrogen to SCr ratio > 20. For aspartate aminotransferase (AST) or alanine aminotransferase (ALT) elevations, mild elevation was defined as an increase in either value of 2 to 5 times ULN; moderate elevation as an increase of 5 to 15 times ULN; and severe elevation as an increase greater than 15 times ULN.

### Statistical analysis

Collected data are presented descriptively, with continuous variables presented as median (interquartile range, or IQR), and categorical variables presented as n (%).

## Results

### Patient population

**Fig. 1:**
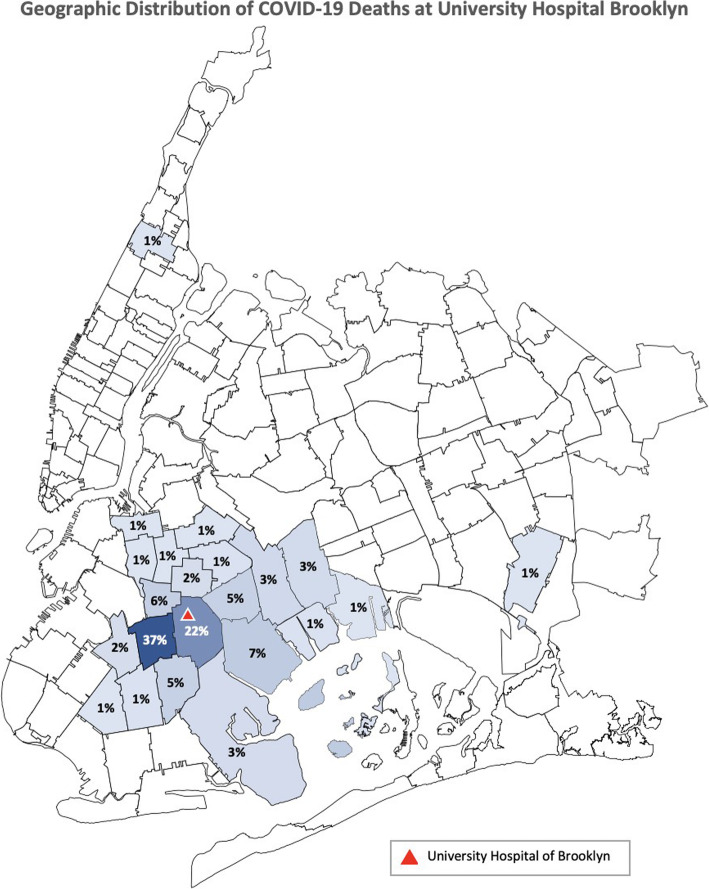
Distribution of COVID-19 deaths by ZIP code in New York City, New York

Two hundred expired, confirmed COVID-19 positive patients were reviewed for this study, consecutively enrolled from the date of first in-hospital expiration. Characteristics of the 200 expired patients are provided in Table 1. Our study population was largely male (58·5%), Black American (89%) Brooklyn residents. Sixty-nine percent were from East Flatbush, followed by Canarsie and Bedford Stuyvesant/Crown Heights (10 and 9% respectively). Two patients reviewed were from outside Brooklyn – one from Queens and one from Central Harlem. (See Fig. 1 for geographic distribution data.) Eighty-five percent of patients were 60 years of age or older, and almost one-fourth (24·5%) between the ages of 80 and 89, with a median age of 73 years (IQR 62–82). Most patients presented from the community (89·5%), with 10·5% presenting from local nursing facilities in Brooklyn. Eleven of our patients presented to UHB for reasons other than COVID-19, but subsequently tested positive and expired due to complications consistent with the viral process.

**Table 1:** Baseline characteristics, *n* = 200 patients

Age in years, median (IQR)	73 (62–82)
Breakdown by age group (*n,* %)	
≤ 39	3 (1·5)
40–59	26 (13)
60–79	107 (53·5)
80–89	49 (24·5)
> 89	15 (7·5)
Male (*n,* %)	117 (58·5)
Race (*n,* %)	
Black	178 (89)
Latino	7 (3·5)
White	4 (2)
Undisclosed	11 (5·5)
Origin (*n,* %)	
Home	179 (89·5)
Nursing home	21 (10·5)
Admitted to hospital for reasons other than COVID-19 (*n,* %)	11 (5·5)
Weight	
TBW, median (IQR), kg	77.6 (68·55–90·7)
BMI, median (IQR), kg/m^2^	29 (25·1–32·9)
Breakdown by BMI (*n,* %)	
Underweight (< 18.5)	4 (2)
Normal weight (18.5–24.9)	45 (22·5)
Overweight (25–29.9)	68 (34)
Obese (≥ 30)	83 (41·5)
Past Medical History (*n,* %)	
Hypertension	169 (84·5)
Diabetes mellitus	115 (57·5)
Hypertension and diabetes mellitus	101 (50·5)
Obesity	83 (41·5)
Coronary artery disease	49 (24·5)
Chronic kidney disease or end-stage renal disease	40 (20)
On hemodialysis prior to admission	14 (7)
Cerebral vascular disease	28 (14)
Asthma	16 (8)
COPD	17 (8·5)
History of malignancy	12 (6)
HIV-positive	7 (3·5)
Renal transplant	4 (2)
Known current or former smoker	19 (9·5)
History of ACE inhibitor/ARB use	59 (29·5)
No known past medical history, not obese	13 (6·5)
Number of comorbidities^a^ (*n,* %)	
No comorbidities	10 (5)
1 comorbidity	28 (14)
2 comorbidities	52 (26)
3 comorbidities	54 (27)
4 or more comorbidities	56 (28)
More than 1 comorbidity	162 (81)
Three or more comorbidities	110 (55)

^a^including obesity as a comorbidity

Analysis of past medical history found hypertension (84·5%) and diabetes mellitus (57·5%) to be the most common comorbidities among this population, and half (50·5%) of patients had history of both. Obesity was the third most common comorbidity, with 41·5% of patients having calculated BMI ≥30. Patients with history of coronary artery disease (CAD), chronic kidney disease (CKD) or end-stage renal disease (ESRD), and cerebrovascular disease (CVD) represented 24·5, 20, and 14% of our study population. Overall, 95% of patients had at least one comorbidity, 81% had more than one, and 55% of patients had three or more comorbidities.

### Patient presentation

Table 2 outlines the admission characteristics of patients at the time of hospitalization. The most common symptoms consistent with COVID-19 reported on admission were dyspnea or shortness of breath, followed by cough and fever. One-fourth of patients presented with all three symptoms (dyspnea, cough, and fever), and only 17% reported dyspnea alone. In these patients, 7% reported known COVID-19 positive contacts, and none reported international travel in the past 6 months. The median time from onset of symptoms to workup for COVID-19 was 4·45 days (IQR 2–7).

**Table 2:** Admission characteristics, *n* = 200 patients

Symptoms associated with COVID-19 (*n,* %)	
Dyspnea	185 (92·5)
Cough	108 (54)
Fever (≥37.3 °C)	108 (54)
Cough, dyspnea, and fever	51 (25·5)
Only dyspnea (no cough, no fever)	34 (17)
GI symptoms	28 (14)
None of the above symptoms	0 (0)
Diabetic ketoacidosis	19 (9·5)
Known COVID-19 positive contact (*n,* %)	14 (7)
Recent international travel (*n,* %)	0 (0)
Duration of symptoms prior to admission, median (IQR), days	4·45 (2–7)
1 to 3 days of symptoms (*n,* %)	102 (51)
4 to 7 days of symptoms	79 (39·5)
More than 1 week of symptoms	19 (9·5)

Laboratory data on admission are presented in Table 3. Forty out of 200 patients had documented CKD or ESRD. In the remaining 160 patients, the median serum creatinine (SCr) on admission was found to be 1·5 mg/dL (IQR 1·2–2·2), and 102 presented with a SCr above ULN (i.e., > 1·3 mg/dL) (63·6%). Elevations in liver function tests (AST or ALT) were present in 65 of 199 patients with available data: 50 with mild elevations, 10 with moderate, and 5 with severe. Lymphocytopenia was present in 51·4% of patients, with a median absolute lymphocyte count of 800 (IQR 600–1000). Elevated inflammatory markers were consistent across the population: median CRP, LDH, and ferritin were above their ULN at 209 (IQR 129·8–281·8), 541 (IQR 407–738), and 1102·4 (IQR 488·9–2619·3) respectively. Median D-dimer and fibrinogen on admission were 2091 (IQR 500–6458) and 482·5 (IQR 460·3–651·8) respectively. See Table 4 for available laboratory data within 24 h of expiration.

**Table 3:** Baseline lab values on admission

White blood cells (WBC) in K/mcL, median (IQR) [ref: 3.5–10.8] (*n* = 199)	8·59 (6·49–11·28)
WBC > 10·8 K/mcL (*n,* %)	54 (27·1)
Absolute lymphocyte count in cells/mcL, median (IQR) [ref: 900–2900] (*n* = 183)	800 (600–1000)
Lymphocytopenia (< 900 cells/mcL) (*n,* %)	94 (51·4)
Serum creatinine (SCr) in mg/dL, median (IQR) [ref: 0.7–1.3] (*n* = 160^a^)	1·5 (1·2–2·2)
SCr > 1·3 mg/dL (*n,* %)	102 (63·75)
Occurrence of acute kidney injury during admission (*n,* %)	128 (80)
Aspartate aminotransferase (AST) in units/L, median (IQR) [ref: 13–39] (*n* = 199)	55 (37–88)
AST > 39 units/L (*n,* %)	142 (71·4)
Alanine aminotransferase (ALT) in units/L, median (IQR) [ref: 7–52] (*n* = 199)	29 (17–44)
ALT > 52 units/L (*n,* %)	36 (18·1)
Elevation of AST or ALT on admission (*n* = 199)	
Mild (2–4.9 x ULN) (*n,* %)	50 (25·1)
Moderate (5–14.9 x ULN)	10 (5)
Severe (> 15 x ULN)	5 (2·5)
C-reactive protein (CRP) in mg/L, median (IQR) [ref: 0–8] (*n* = 164)	209 (129·8–281·8)
CRP > 8 mg/L (*n,* %)	164 (100)
Lactate dehydrogenase (LDH) in units/L, median (IQR) [ref: 140–271] (*n* = 160)	541 (407–738)
LDH > 271 units/L (*n,* %)	151 (94·4)
Ferritin in ng/mL, median (IQR) [ref: 14–233] (*n* = 157)	1102·4 (488·9–2619·3)
Ferritin > 233 ng/mL (*n,* %)	145 (92·4)
Erythrocyte sedimentation rate (ESR) in mm/hr., median (IQR) [ref: 0–20] (*n* = 44)	101·5 (60·3–127·8)
ESR > 20 mm/hr. (*n,* %)	44 (100)
Fibrinogen in mg/dL, median (IQR) [ref: 162–379] (*n* = 16)	482·5 (460·3–651·8)
Fibrinogen > 379 mg/dL (*n,* %)	15 (93·8)
D-dimer in ng/mL, median (IQR) [ref: < 500] (*n* = 59)	2091 (500–6458)
D-dimer > 500 (*n,* %)	44 (74·6)

^a^excluding 40 patients with baseline chronic kidney disease (CKD) or end-stage renal disease (ESRD)

**Table 4:** Lab values on expiration

White blood cells (WBC) in K/mcL, median (IQR) [ref: 3.5–10.8] (*n* = 173)	12·14 (8·84–17·51)
WBC > 10·8 K/mcL (*n,* %)	100 (57·8)
Absolute lymphocyte count in cells/mcL, median (IQR) [ref: 900–2900] (*n* = 140)	800 (500–1100)
Lymphocytopenia (< 900 cells/mcL)	87 (62·1)
Serum creatinine (SCr) in mg/dL, median (IQR) [ref: 0.7–1.3] (*n* = 147)	2·2 (1·25–5·25)
SCr > 1·3 mg/dL (*n,* %)	108 (73·5)
Aspartate aminotransferase (AST) in units/L, median (range) [ref: 13–39] (*n* = 170)	63 (41–129)
AST > 39 units/L (*n,* %)	134 (78·8)
Alanine aminotransferase (ALT) in units/L, median (IQR) [ref: 7–52] (*n* = 170)	35·5 (20–68)
ALT > 52 units/L (*n,* %)	53 (31·2)
Elevation of AST or ALT on expiration (*n,* %)	
Mild (2–4.9 x ULN)	41 (24·1)
Moderate (5–14.9 x ULN)	15 (8·8)
Severe (> 15 x ULN)	15 (8·8)
Lactate dehydrogenase (LDH) in units/L, median (IQR) [ref: 140–271] (*n* = 29)	645 (522–946)
LDH > 271 units/L (*n,* %)	29 (100)
C-reactive protein (CRP) in mg/L, median (IQR) [ref: 0–8] (*n* = 27)	300 (227·5–337·5)
CRP > 8 mg/L (*n,* %)	27 (100)
Ferritin in ng/mL, median (IQR) [ref: 14–233] (*n* = 31)	2367·7 (516·6–3502·7)
Ferritin > 233 ng/mL (*n,* %)	31 (100)

### In-hospital management

In-hospital management is detailed in Table 5. Median length of hospital stay prior to expiration was 4·25 days (IQR 2·26–6·93), with a median total duration of symptoms from onset to expiration of 8·42 days (IQR 5·57–12·72). Approximately, two-thirds of patients expired more than 7 days after onset of symptoms (63·5%). Given our hospital’s designation as a COVID-19-only facility, patients were managed throughout our hospital, with only 27.5% of patients receiving care within our intensive care units – all other patients received care in wards which previously managed patients of lower acuity.

**Table 5:** In-hospital management (*n* = 200)

Length of hospital stay^a^; median (IQR), days	4·25 (2·26–6·93)
Total duration of symptoms prior to expiration,^b^ median (IQR), days	8·42 (5·57–12·72)
Expiration more than 7 days following symptom, (*n,* %)	127 (63·5)
Code Status (*n,* %)	
Full code until expiration	113 (56·5)
DNR order in place at admission	50 (25)
Made DNR during admission	37 (18·5)
Made DNR following intubation	11
Total ICU Admissions (*n,* %)	55 (27·5)
Intubated during stay (*n,* %)	70 (35)
Intubations performed in emergency department (ED) (*n* = 70) (*n,* %)	30 (42·9)
Time to intubation if not intubated in ED, median (IQR) (*n* = 40), hours	57 (35–96)
O2 therapy prior to intubation if not intubated in ED (*n* = 40)	
Nasal cannula (*n,* %)	19 (47·5)
Non-rebreather	13 (32·5)
BIPAP	6 (15)
CPAP	1 (2·5)
Room air	1 (2·5)
Prone positioning (*n,* %)	1 (0·5)
Anticoagulation	
Thromboprophylaxis	
Heparin (*n,* %)	102 (51)
Enoxaparin	59 (29·5)
Fondaparinux	1 (0·5)
Full dose anticoagulation	
Heparin or enoxaparin	11 (5·5)
Continued home medications	10 (5)
No anticoagulation	
Length of stay too short	10 (5)
Elevated INR	3 (1·5)
History of GI bleed	3 (1·5)
Comfort measures only	1 (0·5)
Hemodialysis new start, *n* = 186 (*n,* %)^c^	18 (9·7)

^a^prior to expiration, adjusted to account for patients who were admitted to the hospital for a primary complaint other than symptoms associated with COVID-19 but were found to be COVID-19-positive during their stay

^b^calculated by combining total duration of symptoms prior to concern for COVID-19 and total length of stay from time of concern for COVID-19 to expiration in hospital

^c^excluding patients who were on hemodialysis prior to admission

Of 200 patients reviewed, 113 (56·5%) were designated full code for the duration of their stay, with 43·5% of patients designated Do Not Resuscitate (DNR) at time of expiration. DNR orders were in place on admission for 50 patients (25%), and palliative care discussions moved 37 patients who were full code on admission to DNR, 11 of which occurred after intubation.

Seventy of our patients were intubated (35%), with 30 patients intubated in the field or upon arrival to the emergency department (ED). For the remaining 40 intubated patients, median time to intubation was 57 h (IQR 35–96). Most patients received oxygen supplementation via nasal cannula or non-rebreather prior to intubation (47·5% and 32·5% respectively). Only one patient received prone positioning.

Anticoagulation therapy was utilized in 183 out of 200 patients (91·5%). Most (81%) received thromboprophylaxis – either prophylactic heparin, enoxaparin, or fondaparinux. Those not receiving thromboprophylaxis either received full anticoagulation with heparin or enoxaparin (11, 5·5%) or home medications (10, 5%). Those who did not receive anticoagulation fell into one of four groups: short duration of stay, elevated international normalized ratio, recent history of gastrointestinal bleed, or comfort measures only.

A majority of patients received additional cultures during their stay (see Table 6 for additional culture data). Thirty-five patients had positive culture data – most commonly, suspected contamination with coagulase-negative *Staphylococcus* spp. on blood cultures, followed by *Staphylococcus aureus* (both methicillin-sensitive and -resistant) on blood cultures and tracheal aspirates. Regardless of culture data, most patients (55·5%) received 72 h or more of antibiotic therapy – most often regimens targeted toward community- or hospital-acquired pneumonia (e.g., combinations of ceftriaxone and azithromycin, or vancomycin and piperacillin-tazobactam). Of note, only 21 respiratory viral panels were performed throughout this study period due to shortage of supplies which overlapped with SARS-CoV-2 testing supplies. Only one respiratory viral panel returned positive for human coronavirus HKU1.

**Table 6:** Culture data and antibiotic management

Viral panels, *n* = 21	
Positive (*n,* %)	1 (4·8)
Result	Coronavirus HKU1
Cultures	
Additional cultures drawn (*n* = 200)	179 (89·5)
Positive cultures	35
Blood	
Coagulase-negative *Staphylococcus* spp.	16
Methicillin-resistant *Staphylococcus aureus*	4
*Candida albicans*	2
Methicillin-susceptible *Staphylococcus aureus*	1
*Klebsiella pneumoniae*	1
Tracheal aspirate	
Methicillin-susceptible *Staphylococcus aureus*	2
*Pseudomonas aeruginosa*	2
Methicillin-resistant *Staphylococcus aureus*	1
*Klebsiella pneumoniae*	1
*Enterobacter gergoviae*	1
Urine	
*Pseudomonas aeruginosa*	2
Extended-spectrum beta-lactamase *Escherichia coli*	1
*Klebsiella pneumoniae*	1
Receipt of ≥72 h of antibiotics (*n* = 200) (*n,* %)	111 (55·5)

In addition to standard supportive care, a portion of patients received investigational agents such as hydroxychloroquine and tocilizumab, as well as stress-dose corticosteroids and anticoagulation for thromboembolic events associated with COVID-19 (see Table 7). Hydroxychloroquine was utilized in 134 patients (67%). Median time from viral sample to initiation of treatment with hydroxychloroquine was 16 h (IQR 6–39·75). No patients in this study received remdesivir or convalescent plasma.

**Table 7:** COVID-19 management (*n* = 200)

Pharmacotherapy treatment for COVID-19 (*n,* %)	
Hydroxychloroquine^a^	134 (67)
Full dose anticoagulation^a^	11 (5·5)
Tocilizumab^a^	10 (5)
Steroids^a^	10 (5)
Time to treatment with hydroxychloroquine, median (IQR), hours	16 (6–39·75)

^a^Data presented not mutually exclusive, percentages will not add up to 100

## Discussion

This case series describes 200 patients with laboratory-confirmed COVID-19 infection who died between March 17 and April 16, 2020. No patients had history of international travel, and the majority of patients had no known exposure to COVID-19 positive contacts, making exposure most likely to be community-based. The median age in our case series was 73 years (IQR 62–82), consistent with reports that older persons are at higher risk of dying from COVID-19 infection [10, 14]. In our population, 58·5% of patients were male, consistent with other reports that males have higher risk of death than females as they may manifest more severe disease [15].

In our case series, 178 out of 200 (89%) patients were Black Americans. The COVID-19 pandemic has highlighted the disparities pervasive in our healthcare system. Data from the Centers for Disease Control and Prevention describe disproportionate burden of COVID-19 illness and death among racial minorities [16]. In NYC, the COVID-19 fatality rate in Black Americans is double that of white patients [8]. The excessive burden of COVID-19 on racial and ethnic minorities is multifactorial and includes, but is not limited to, vast inequalities in socioeconomic status, education, physical environment, social support networks, and access to health care.

More than two-thirds of our patients were residents of the East Flatbush neighborhood of Brooklyn. East Flatbush, along with the surrounding neighborhoods also represented in this study, is a predominantly Black and foreign-born community [17]. Residents of these communities are more likely to live in multigenerational homes, commute using public transportation, and are less likely to receive paid sick leave – all factors which may contribute to increased rates of SARS-CoV-2 transmission [18]. The median household income for East Flatbush is $48,000, compared to $77,000 for the borough of Manhattan, with 42% of East Flatbush residents making less than $25,000 per year. This financial burden translates to limited access to health care and poorer overall health. East Flatbush ranks 12th out of 59 NYC neighborhoods for percentage of adults without health insurance (15%) [18]. The COVID-19 pandemic has demonstrated that, during times of crises, health disparities are exacerbated and serve to weaken already disadvantaged and vulnerable populations.

More than half of our patients had three or more comorbidities, with hypertension, diabetes, and obesity being the most common. This is consistent with previous data showing that Black Americans experience higher rates of comorbidities compared to white Americans [19]. Only 33 patients in our study had a history of asthma and chronic obstructive pulmonary disease, and similar underrepresentation of both diseases has been reported elsewhere. Several factors have been postulated [20], including poor recognition of chronic respiratory disease due to overlapping of symptoms with COVID-19 and potential protection against COVID-19 by chronic respiratory disease. In addition, therapies used by these patients may reduce risk of infection or development of symptoms leading to diagnosis of COVID-19.

Dyspnea was the most common presenting symptom (92·5%) – a prevalence not seen in recent data from the US and China [10, 21, 22]. This likely highlights the level of severity of illness in our patients at presentation. Similar to other studies, more than 50% of our patients had cough and fever [10, 14]. Interestingly, approximately 10% of our patients presented in diabetic ketoacidosis. It is unclear if this is directly related to pathogenesis of COVID-19 or non-adherence to medications: about 50% of our patients had more than 4 days of COVID-19 symptoms prior to admission, and it is possible that patients, while sick at home, may not have continued their maintenance medications.

AKI is well-documented in viral infections, such as severe acute respiratory syndrome (SARS) and Middle East respiratory syndrome-related coronavirus (MERS-CoV), with estimates ranging between 5 and 15% of cases, and AKI and has been identified as a risk factor for increased mortality [23]. Available data from China [24, 25] did not identify high rates of AKI, and only small portions of patients in these studies expired. The high rate of AKI (80%) in our population may implicate AKI as a potential indicator for the severity of COVID-19 infection.

Cytokine release syndrome, a state characterized by worsening respiratory failure and markedly elevated inflammatory markers such as CRP, LDH, and ferritin, is a frequent consequence of COVID-19 infection, similar to SARS and MERS-CoV [26]. Our findings of elevated CRP, LDH, and ferritin in almost all of our patients at baseline indicate the severity of their presentation, and raise a consideration of these laboratory values as markers of prognosis in COVID-19. CRS often results in mechanical ventilation, and 70 of our 200 patients received mechanical ventilation during their admission, nearly half of whom were intubated on presentation. CRS is thought to peak approximately 8 days after symptom onset [26]. Our patients had a median duration of symptoms – from time to symptom onset to expiration – of 8·42 days (IQR 5·57–12·72), and inflammatory markers indicative of CRS were elevated in all patients with available data at expiration. The role of CRS management in COVID-19 remains unclear.

Severe COVID-19 infection has been associated with increased risk of thromboembolic events. Data show an estimated 16·7% [27] to 25% [28] of patients experience venous thromboembolism during their admission. D-dimer and fibrinogen were elevated in 74·6% and 93·8% of our patients with available data, consistent with reports from France [27]. Most of our patients received thromboprophylaxis, and 11 out of 200 patients received therapeutic anticoagulation for new-onset thromboembolic events. Further data are needed to identify the benefit of full-dose anticoagulation therapy in patients with COVID-19 compared to standard thromboprophylaxis.

Only one of our patients received prone positioning, an intervention shown to reduce mortality if initiated early in patients with severe acute respiratory distress syndrome [29]. At the time of data collection, prone positioning was infrequently performed at UHB, and teams have since been established to coordinate prone positioning for COVID-19 patients in severe respiratory distress.

As of late April 2020, there are no approved agents for the treatment of COVID-19 infection. Supportive management remains the standard of care, as in other viral pneumonias. Based on preliminary investigational data [30, 31] showing potential benefit, two-thirds of our patient population received hydroxychloroquine during their stay. Few of our patients received corticosteroids, likely given the inconclusive data to support their use in COVID-19 available during the study period. Additionally, a small group of patients received tocilizumab, an interleukin-6 receptor antagonist, which has been considered in COVID-19 patients in an effort to reduce the inflammation associated with CRS. The true benefit of these agents has yet to be determined.

In non-COVID-19 related therapy, 55·5% of patients received 72 h or more of antibiotic therapy. Very few patients were found to have culture-confirmed infectious processes other than COVID-19 during their stay, and even fewer had positive respiratory cultures. It remains unknown the exact rate of bacterial coinfection in patients with COVID-19.

Our study is not without limitations. This descriptive observational study was retrospective in nature and lacked a control group. In an effort to reduce selection bias, patients underwent consecutive enrollment, and were only included in analysis if they had laboratory-confirmed diagnosis of COVID-19. Also, inconsistent collection of laboratory values such as CRP, LDH, ferritin, D-dimer, and fibrinogen limited the number of patients in whom these values could be evaluated. Since the data collection period, these values have become part of the standard COVID-19 workup at UHB, both at baseline and trended throughout admission. Finally, lack of robust data such as randomized trials on the novel coronavirus presents difficulty in comparing our observations with those of others, particularly given differences between our patient population and that of other countries reporting on COVID-19.

To our knowledge, this is the largest description of patients who died due to COVID-19 at this time. UHB serves a majority Black American population, with a large portion of patients of Caribbean descent, who have multiple comorbidities. Considering the racial, economic, and healthcare disparities of Brooklyn, multiple systematically-based factors – modifiable and non-modifiable – may contribute to our patients’ baseline characteristics and severity of illness on presentation, which ultimately resulted in their mortality.

Available literature on COVID-19 continues to evolve. Description of the demographics and presentation characteristics of patients who have expired due to COVID-19 identifies populations at high risk of severe illness and ultimate mortality, and assists providers in triaging and caring for COVID-19 positive patients.

## Conclusions

Given the lack of data available on the backgrounds and presentations of Black American patients with COVID-19, we present an observational data set of patients who expired in-hospital, in hopes of improving the care of these patients at high risk of mortality.

## Data Availability

The datasets used and analyzed during the current study are available from the corresponding author on reasonable request.

## Abbreviations

AKI: Acute kidney injury
AST: Aspartate aminotransferase
ALT: Alanine aminotransferase
BMI: Body mass index
CKD: Chronic kidney disease
COVID-19: Novel coronavirus disease 2019
CRP: C-reactive protein
CRS: Cytokine release syndrome
ESR: Erythrocyte sedimentation rate
ESRD: End-stage renal disease
IQR: Interquartile range
LDH: Lactate dehydrogenase
MERS-CoV: Middle East respiratory syndrome-related coronavirus
SARS: Severe acute respiratory syndrome
SARS-CoV-2: Severe acute respiratory syndrome coronavirus 2
SCr: Serum creatinine
SUNY: State University of New York
NYC: New York City
UHB: University Hospital of Brooklyn
ULN: Upper limit of normal
US: United States
WBC: White blood cell count
